# From Guidelines to Lifelines—An Ethnographic Study of How Diabetes Management Is Emplotted During Clinical Encounters with Young Adults with Type 1 Diabetes

**DOI:** 10.3390/healthcare13040374

**Published:** 2025-02-10

**Authors:** Signe Hellung Schønning, Ayo Wahlberg, Eva Hommel, Dan Grabowski

**Affiliations:** 1 Steno Diabetes Center Copenhagen, Borgmester Ib Juuls Vej 82, 2730 Herlev, Denmark; eva.hommel@regionh.dk (E.H.); dan.grabowski@regionh.dk (D.G.); 2 North Zealand University Hospital, Dyrehavevej 29, 3400 Hillerød, Denmark; 3 Institute of Anthropology, Faculty of Social Sciences, University of Copenhagen, Øster Farimagsgade 5, 1353 Copenhagen, Denmark; ayo.wahlberg@anthro.ku.dk

**Keywords:** emerging adults, type 1 diabetes, clinical encounters, chronic illness, therapeutic emplotment

## Abstract

**Background/Objectives:** Working with young adults with T1D in outpatients clinic entails achieving a delicate balance between maintaining trust and improving diabetes management. By looking at the interactions between healthcare professionals and young adults with T1D as narrative emplotment, this article seeks to investigate how illness narratives are part of and actively worked on in consultations. **Methods:** Based on ethnographic observations of fourteen consultations with young adults 18–23 years of age, three narrative strategies to promote better diabetes management among the young adults were identified: (1) replacing sub-optimal practice with technology, (2) encouraging enhanced autonomy, and (3) setting realistic standards for diabetes care. Each strategy works to create a meaningful explanation for experienced challenges, formingas a basis for improved diabetes self-management. **Results:** Consultations were found to create a space where the meaning of living with an illness can be discussed between the healthcare professionals and the patients. **Conclusions:** Looking at how this meaning is negotiated in the consultations is an important aspect of understanding how daily diabetes management is made practicable, especially when working with young adults who are constituting their identities and often live with sub-optimal glycaemic control.

## 1. Introduction

In Denmark, type 1 diabetes (T1D) is one of the most common chronic illnesses in childhood, with a slight increase in the number of people diagnosed with T1D in recent years, as well as a shift in the time of onset, such that more are being diagnosed under 20 years of age [1,2]. Adolescents and young adults are especially prone to living with higher levels of blood glucose, which over a lifetime may lead to diabetes complications [3]. At the same time from a clinical perspective, a rise in psychological challenges can be observed during late adolescence, with higher rates of depression, anxiety, substance abuse, and eating disorders reported among people with diabetes compared to their peers [4]. Many barriers to optimal diabetes management exist during emerging adulthood and late adolescents, ranging widely from social factors to therapy related factors [5]. At the same time, adolescence is a time where identities are constituted, and navigating a chronic illness during identity formation can be challenging both in terms of illness identity and glycaemic control [6]. As the illness frequently debuts at an early age, people with T1D have often lived with the condition for a long time as they approach adulthood. When facing the transition into adulthood, diabetes management tasks are already familiar to the young adults, and they often possess the knowledge needed to calculate carbs and regulate high or low blood glucose in everyday life. Yet how specific care tasks are carried out and how they are shaped changes over time [7]. This article uses a narrative phenomenological approach to understanding interactions between patients and healthcare professionals in the clinic [8]. The aim is to understand how healthcare professionals and young adults living with T1D work together to ascribe meaning to diabetes management actions. As an ethnographic study, we investigate how these strategies are evident in everyday interactions at a diabetes outpatient clinic. Anthropological and ethnographic studies seek to challenge the taken-for-granted nature of everyday life and assumptions concerning how we live our lives [9]. Hospital ethnography has the potential to look into why hospitals are not solely sites of of biomedical practicewith significant differences in praxis [10]. This study has sought to map out narrative strategies deployed to motivate young adults to improve their diabetes self-management. The study has been conducted at a diabetes outpatient clinic in Greater Copenhagen where biweekly afternoons are reserved for appointments with emerging adults. The first author has been present during these afternoon appointments, attending consultations to investigatethe strategies employed to motivate emerging adults with T1D towards better self-management.

## 2. Materials and Methods

As a condition-proximate outpatient ethnography [11], the present study focus on how T1D and T1D regulation are talked about with young adults, particularly during consultations. To study illness narratives, observations were carried out at a diabetes outpatient clinic throughout a 3-month period from September to December 2021. A total of 14 consultations were observed with emerging adults (18–23 years old). Between consultations or when a patient did not show up, the time present at the outpatient clinic was spent engaging in informal conversations with patients and healthcare professionals. Informal conversations are beneficial at the beginning of a period of fieldwork, as they help identify what is at stake for the actors in the field. Through informal conversations, the researchers build rapport by investing time in the field and becoming familiar with the (often unspoken) norms and rules of the field [12]. A total of 8 informal consultations with a minimum duration of 20 min were carried out and extensive field notes were taken. The informal conversations were with emerging adults aged 18–32 and, in three cases, with their mothers.

Informants were recruited in the clinic’s lobby while they were waiting for their next appointment. They were asked whether they would be interested in participating in the research. Prior to the consultation, informants gave their verbal consent to participate in the study and afterwards written consent was obtained. Here, the informants had the opportunity to ask in-depth questions concerning the research project. The young adults were then asked whether they wanted to engage further in the research process by participating in an interview. A total of seven interviews were carried out with young adults with T1D to generate a deeper understanding of their experiences of living with T1D during emerging adulthood.

The first author jotted fieldnotes during and between consultations, typing them into a computer shortly after where further details were added. Notes from all consultations were then read through, and once familiarized they were translated into codes, further condensed into units of meaning, through the process of systematic text condensation [13]. The initial coding was performed by the first author (SHS). To enhance rigour, the findings were discussed with authors DG and AW and pitched to healthcare practitioners who carry out consultations with emerging adults to see if they resonated with their practice. These units of meanings are the foundation of the three narrative strategies depicted in the article. In the writing process, the three consultations presented in this paper were selected for an in-depth narrative analysis, following narrative phenomenology and therapeutic emplotment [8,14]. The material contained multiple examples of the strategies in practice, often used in combination. The three cases all exhibited one exemplary strategy and were therefore chosen to present the given narrative strategy.

### Theoretical Framework

By using Cheryl Mattingly’s (1994) theory of therapeutic emplotment, we can investigate narratives as they unfold during the clinical encounter. Mattingly uses the concept of therapeutic emplotment to analyse how health care professionals actively work to shape therapeutic acts into a coherent plot by enfolding clinical encounters into larger developing narrative structures. Thus, actions demand that we plot and that actors are motivated to create stories while acting. Making sense of a situation can happen by shaping a narrative structure—with a beginning, middle, and end—around actions that work toward improving the situation. Among many others, Mattingly provides the example of an occupational therapist who is working on a patient with a severe brain injury that has awakened from a coma. The occupational therapist hands the patient a comb and asks him to comb his hair. When the patient does not want to do so the therapist adds meaning to the action by saying that it is a good balance exercise. He then finally combs his hair and gains courage to engage in further activities that serve not only to improve his condition but also to encourage him to reintegrate into society [14]. The clinical encounter provides a space for emplotment and enables the reconfiguration of a coherent and meaningful narrative, which incorporates actions of caring for the illness and improving the condition [14]. In what follows, we will mobilize the theory of therapeutic emplotment to shed light on the narrative strategies employed by healthcare professionals working with young adults with T1D. Mattingly’s theory allows us to investigate how illness narratives are shaped between patient and health care professionals.

These strategies take shape through narrative emplotment emerging between the practitioner and the patient in the consultation. Illness narratives differ from biomedical explanationsin the sense that they are actor centred rather than diseasecentred. As humans, our actions are structured around the sense of an ending, thus our actions are directed in a way that is intended to bring about the desired ending [14]. In a clinical setting where chronic illness and suffering often generate a narrative of loss, the life story of patients has to be rewritten if it is to accommodate the bodies disabled by illness [14]. According to Mattingly, a moment of therapy is an enriched present, where what really matters is what the moment portends for the future, rather than what is happening in the moment. In that sense, therapy and narrative genres can produce hope for patients and relatives living with a chronic condition. Hope then becomes a process of generating lives that are worth living [8]. Thus, investigating narratives that emerge in the consultation room offers a means to analyse how hope for a better future is created among emerging adults with T1D and their healthcare professionals.

For young adults, who are in the process of constituting their identities, the narrative framework demands a certain flexibility as the future, for many, is uncertain. James Marcia’s concept of identity, as a strong sense of continuity with the past, an active direction in the present, and future trajectory [15], corresponds with the notion of a narrative as a coherent story connecting one’s past, present, and future. Likewise, narratives are stories of life lived in the middle of an unfolding present that are connected to a past and a future [8]. The stories the emerging adults tell about their life with T1D can be seen in relation to how they perceive their past with the condition, how they experience their present, and where they see themselves in the future.

## 3. Results

As the first author was present during the consultations, she was also occasionally addressed directly from time to time, which also applies to the empirical cases presented throughout the findings. The findings are presented with a focus on each of the narrative strategies we identified, emphasizing how the healthcare professionals work with young adults to create a meaningful story around actions that will improve their diabetes management. When asked directly, healthcare professionals at the diabetes outpatient clinic say they work toward getting young adults to take responsibility for their own diabetes management and, thereby, to become well regulated. We used pseudonums for the people presented in the cases in this article. As we will see, through applying a narrative phenomenological framework, it is possible to interpret how healthcare professionals work to construct a meaningful story around diabetes management.

### 3.1. Replacing Suboptimal Practices with Technology

One of the strategies commonly employed to emplot a better future with diabetes is to invoke existing technological meanst to assist in monitoring and regulating one’s diabetes.

*A young man, Peter, enters the consulting room of Mie, an experienced diabetes nurse. He is 19 years old and wears a thin black down jacket with a silver logo on the back. This consultation is his first with a nurse in the department as he just transitioned out of pediatric care. As Mie greets him she reads from his journal that he has an insulin pump and asks if he likes it. He answers “it works” without changing his tone of voice.*

*Mie: And how do you measure your blood sugar?*

*Peter: I’m a ‘finger-pricker’… but it’s not what I do best.*

*Sensing some dissatisfaction in his tone of voice, Mie asks caringly how he feels about his diabetes at the moment.*

*Peter: It’s okay. I had a longer period at the beginning where I didn’t want to take care of my diabetes. I know how to enter [to the pump] and I take insulin at all meals, but I might be a bit tired of it. I always enter, but I don’t always measure.*

*Mie: How come you don’t have a sensor? [CGM]*

*Peter: No one has ever offered me one…*

*Mie: how about I offer you one today? [with a grin on her face]*

*Peter: Is it the one that sits there? [he points on his arm, Mie nods] How many times a day would I have to replace it? [a bit sceptical]*

*Mie: Every 14 days*

*Peter: Okay! [enthusiastically]*

*(…)*

*Mie goes to find a CGM for Peter and begins instructing him on how to apply it and how it works*

*Peter: [asks nervously] Does it hurt? How big is the needle?*

*Mie: I have patients telling me it doesn’t hurt at all.*

*Peter: But I like to know these things, can I see the needle before I apply it? Wow, it’s really nice—every 14 days, that’s twice a month! [Peter looks fascinated at the box the CGM comes in].*

*Mie: Okay, so I will instruct you on how to apply it, and then you can do it yourself.*

*Mie explains to Peter how the sensor works and continues to instruct him. He looks intriguingly at the box. His HbA1c test result comes in, it is 103. Mie highlights the number on her screen but doesn’t mention it.*

*Mie: Now, you have to place it here [points on her arm], it makes a bit of a loud snap when you apply it, but it isn’t too bad.*

*Mie doesn’t say anything else before Peter has pressed and applied the sensor on his arm filling the room with a loud clicking sound that still rings a bit. He turns to the researcher.*

*Peter: I kid you not, I did not feel it at all.*

*Peter has downloaded the app to scan his sensor, but it must calibrate for 20 min before it works. He keeps updating his app to see if it works yet.*

*Mie: [while typing on her computer] So, about you being tired of your diabetes-*

*Peter: I don’t think I’m tired of it, if I was tired of it, I wouldn’t have registered the carbs and I do register them.*

*Peter explains how he has now accepted living with diabetes. When he was younger, he saw a psychologist for a while, because his troubles with having diabetes influenced his relationship with his mom.*

*Peter: it’s simply that when I’m high, I don’t want to measure my blood sugars to see a high number.*

*Mie: But I get a feeling, that you’re a bit tired of yourself?*

*Peter: Every night I tell myself, tomorrow I will measure, but then a thousand things happen, and I have to prepare a car for rental, and then I forget all about it [T1D].*

*Mie: You know, I find it a bit absurd, you seem quite ambitious and then you have these quite high blood sugars that keep you from achieving the maximal outcome from your work.*

When Peter enters the consulting room, he reveals that he does not measure his blood glucose as much as he would like to. His nurse, Mie, notices that he has an issue with his self-management and finds a technical solution for it. She takes the opportunity to offer him a new means of measuring his blood glucose. By introducing a new technology for monitoring blood glucose, she replaces the already existing infrastructure around it. For Peter, the new CGM addresses his issues with monitoring his blood glucose, simply by constantly monitoring it for him. By doing so, it alleviate the issues he has with his diabetes management and allows him to start over by configuring new practices.

Not only does Mie work on the practices, but she also emplots on the meaning connected to his practice. Prior to the consultation, Peter saw the act of measuring his blood glucose as a confrontation with the fact that he was not doing enough to manage his diabetes. He would feel that his blood glucose might be on the high side but was not motivated to face the fact that it was, causing him to avoid measuring. The act of measuring blood glucose was, for Peter, connected to feelings of guilt and of not being good enough.

By sharing with Peter her impression that he was quite ambitious and by wondering why he allows himself to be held back by his relatively high blood glucose levels, Mie emplots a new story of maintaining and monitoring his blood glucose for his own ambition’s sake. The vision of being able to achieve more if his blood glucose improves, combined with the new technology that offers a new future for him and blood glucose monitoring, seems to create a meaningful narrative for him. With the new CGM, it becomes possible for Peter to configure a new future for himself and his blood glucose. Mie not only gives him a technology that makes his past struggles irrelevant for his future diabetes care, but in interacting with Peter, the foundation for a future without having to let himself down is created through narrative emplotment.

Because the technology has replaced his blood glucose monitoring practices, he can no longer feel bad about his own efforts, and the new technology replaces his guilt and sub-optimal regulation practices. In the case of Peter, the strategy of introducing a new technology for diabetes management serves as a tool for rewriting his illness narrative. He can no longer tell the story of not measuring his blood glucose, because now he has a tool that continuously monitors it for him. Mie manages to connect his negative emotions about diabetes with his lack of blood glucose monitoring, thus expressing her concern about him being quite ambitious but having blood glucose levels that prevent him from achieving the maximum outcome of his actions. Being ambitious seems to suit Peter, and thus the task of blood glucose management becomes part of a meaningful narrative. If the narrative emplotment goes well, the task of maintaining a good blood glucose level will be in his own favour instead of being a desire that another person imposes on him.

### 3.2. Encouraging Enhanced Autonomy

Another strategy employed during outpatient encounters is to enhance young adults’ sense of autonomy regarding their own diabetes practice. One way of doing this is by encouraging a patient’s progress in becoming more independent in managing their diabetes. This can be done by emphasizing what the emerging adults already do that is beneficial to their care, as exemplified in the following consultation:

*A tall young man walks along the hallway with his mom. They walk calmly and quietly along the hallway, and you can sense that they’re accustomed to the situation. With a big smile, their diabetes nurse Line says:*

*Line: Hi Thomas, it’s great to see you. This is the first time you and I get to meet, and I’ve been looking forward to it. I can see you brought your mom today?*

*Thomas and his mom nod*

*Line: I’ll be your nurse here at the hospital, and I’ve taken a look at your health record, but what I really want to hear is how you think things are going?*

*Thomas smiles with a small laugh and says that things are going pretty well*

*Line: Is it okay with you, if I ask your mom as well?*

*Thomas: You just go ahead; she always has something to ask about.*

*Mom: It’s hard not to care a bit about it, but it’s also so that we find things have been good lately, so I’m curious to hear if it shows in his numbers. He’s been doing things a bit differently since last time.*

*Line lights up in an enthusiastic smile.*

*Line: That’s a really good question, I just mentioned it to [SHS] right before you came, that here is someone who really takes good care of his diabetes. When I saw your results, I thought “wow, what has he done?” because it really looks good. So, what have you been doing differently since last time?*

*Thomas smiles at the appraisal and casually leans back in his chair in the consultation room*

*Thomas: Well, I just try to keep it [blood sugar] below 10. I work as an electrician, so I’m quite active at work. I used to think it was good to be around 10 at work, when you’re as active as I am, but last time the doctor told me that it might be wise to keep it a bit below, so now I always keep it under 10 and then my pump deactivates around 6 at work.*

*His mother continues the story:*

*Mom: Yes, there was a slight misunderstanding, you thought it was okay to be at 10, but then you learned that it’s even better to be a bit below.*

*Thomas begins speaking again and says:*

*Thomas: So now I prefer to stay between 5 and 7.*

*Line has been listening enthusiastically and says:*

*Line: Well, whatever you’ve been doing, it’s been working great so keep up the good work!*

Prior to the consultation, Line had pointed out to the researcher that she was curious to hear what had happened, because his HbA1c levels had dropped drastically since his last consultation. What becomes clear in the conversation between Line, Thomas, and his mother is that Line works actively to emphasize that it is Thomas’ own actions that have driven that change. Thomas’ HbA1c is still a bit high, but because it has come a lot closer to the target level, Line decides to focus on the positive developments in his self-management.

In Thomas’ consultation, his mother is present and is also part of his daily diabetes management. Line actively lets Thomas decide to what degree his mother can take part in his consultation and gives him the power to set the agenda for the consultation. She addresses Thomas by name but always asks his mother for information through him. This further emplots the narrative that Thomas is the one in charge of his diabetes management. When told that the doctor cleared up a misunderstanding he had regarding his blood glucose levels, Line continues the narrative by mentioning that the changes Thomas has made are the ones that are working.

Line strengthens the narrative by continuously confirming that the numbers from his blood test show that he is on the right track. The measurements from the blood tests taken at the hospital have a lot of authority, as they are interpreted as an indicator of how well-regulated the person with diabetes is. Having lived with diabetes for a long time, Thomas and his mother understand that the tests taken prior to consultations are of importance, and they are eager to find out “what the numbers tell”. Line employs the numbers not by mentioning the exact value but focusing on the improvement that has happened. When doing so, she ascribes the change to Thomas’ own abilities and skills in managing his diabetes. The strategy of enhancing young adults’ own autonomy serves to give patients a sense of control over their diabetes. For Thomas, it is a path toward complete control, whereas for others it is a strategy in identifying the first step toward taking control of their diabetes management.

### 3.3. Realistic Standards for Diabetes Care

Given that there is an understanding among healthcare professionals that young adults are in the process of constituting their identities and visions of the future, there tends to be some flexibility in the standards healthcare professionals hold young adults to in the consultations. This often involves a diversion from the clinical guidelines, where higher levels of HbA1c are accepted than clinically recommended, or overlooking the times the patient has been a no-show and instead praising the fact they came to one appointment. The narrative strategy is portrayed in the following consultation, in which a young woman and her nurse discuss future management plan, with a focus on her HbA1c:

*20-year-old Maja enters the consultation room along with her boyfriend. As she enters the room, she mentions how she sees a new nurse every time and that she would prefer if it was the same nurse every time. Her nurse, Louise, agrees with her and mentions that it is supposed to be the same.*

*Maja was diagnosed with T1D as a 9-year-old and has been in outpatient care ever since. She tells her new nurse about how the healthcare professionals and the ways they have worked with her have been a mixed experience. Because of that, she is very sceptical toward healthcare professionals and has often left the consulting room during the consultations. She has recently gotten a CGM, and explains how she feels that she has more control over her blood glucose with it. Louise and Maja talk about her blood glucose levels from the data provided by the CGM. Her test results come in and they can also address her HbA1c.*

*Louise: Preferably your HbA1c should be between 48–53, but it is generally a bit higher among youths and young adults. I think that it can be okay to be around 60–70, but yours is 87 and that’s too high. You might feel okay right now, but just 5 years with these levels can cause long-term damage.*

*Louise emphasizes that she is in a process of gaining control of her diabetes, and that it is important to stay on that path. Maja has been taking in what Louise says and adds that sometimes she wakes up with a high blood glucose level, which makes her feel ill.*

*Maja: It’s a bad start to the day of you wake up with a headache and have to take a lot of insulin.*

*Louise understands why Maja doesn’t like it, and from the CGM data she can see that it happens quite often. She mentions how waking up with a very high blood glucose level means waking up sick, and she understands how that can lead to sick days from work. They talk about how to prevent it and settle on changing the ratio between the slow-release and fast-release insulin, so she hopefully won’t start as many days with high blood glucose.*

*As the consultation is about to wrap up, Maja turns towards the researcher and says:*

*Maja: This is a really good nurse; I hope you write that down. There aren’t a lot of them, and it really matters. I’ve left consultations earlier when I haven’t felt welcome or understood, and it’s just so nice to feel that you are understood. Sometimes having diabetes makes you feel all alone in the world.*

When Maja walks into the room, she shares the news of her excitement over her CGM as well as her scepticism toward the healthcare system by saying that she is not fond of constantly having to see new nurses. Her nurse, Louise, interprets her excitement as a sign of progress and enthusiasm in relation to better diabetes management, noticing that Maja is sceptical toward healthcare professionals. By agreeing with Maja that always changing personnel is far from optimal, Louise writes herself into the position of a like-minded ally in Maja’s diabetes management. This action is important in enabling Louise to encourage Maja’s development toward better diabetes management. Louise must be able to gain Maja’s trust if she is to emplot a future with her.

As is clear from the consultation, the HbA1c test results are important in planning Maja’s next step toward better diabetes management. After the initial conversation, Louise hears that Maja feels like she is getting better control of her diabetes, and she encourages Maja’s progress in maintaining control. An HbA1c of 87 is fairly high and is interpreted as sub-optimal diabetes management by healthcare professionals, but because Maja’s experience was an improvement in her diabetes management, Louise does not interpret it as necessarily sub-optimal diabetes practice but uses the measurement to motivate Maja to actively correct her blood glucose more often. By mentioning that the official clinical guidelines name a target level for HbA1c between 48–53, she provides Maja with the biomedical regime’s guidelines for diabetes management. When discussing Maja’s HbA1c, Louise says that many young adults tend to have a higher HbA1c than recommended, which helps Maja feel like she is not alone with her diabetes. Second, Louise says she thinks it may be acceptable for young adults like Maja to have an HbA1c of 60–70. In doing so, Louise gives Maja a target that seems more attainable than lowering her HbA1c to the recommended levels.

Louise emplots the task of lowering Maja’s HbA1c as an extension of Maja’s own transformation toward having improved control over her diabetes, and because they work together to identify mornings with high blood glucose levels as a good target for action prior to their next consultation, it becomes a task that is both meaningful and feasible. Louise builds on Maja’s own understanding that she is gaining control over her diabetes management, adding that aiming for even lower blood glucose levels is a natural next step for Maja.

In the consultation between Maja and Louise, the numbers also become an authority that interacts with Maja’s own experience of how her diabetes management is improving. The numbers indicate that her management is not as good as it should be and her test result is then mobilized to motivate a change towards tighter diabetes management. For Maja, the standards for her diabetes management are looser than the clinical guidelines, making it easier for her to meet them. This serves as a trust-building strategy, as the lower standards create a sense of individualized care and, thus, make the young adult feel seen as an individual. If Maja had been asked to lower her blood glucose to the recommended levels right away, she might have felt overwhelmed or not seen in the consultation and given up immediately, as she had done in prior consultations. Louise’s lowering of the standard Maja is expected to meet for the next appointment motivates Maja to improve her diabetes management and show up next time. It does not necessarily emplot a fixed future, but it is a strategy that serves to make young adults feel seen and understood in the consultation, which promotes trust in the clinician-patient relation.

## 4. Discussion

The three narrative strategies employed when working with diabetes management among young adults that we have identified all serve to foster greater self-management. When the first strategy, which involves introducing a technological solution to a problem with sub-optimal diabetes practice, is employed, the young adult leaves the consultation relieved that a major problem with diabetes management has been resolved. The new technology at first seems like a quick fix, in that the issue it addresses completely vanishes. Yet technology often needs to be tamed and adjusted to everyday life so that it does not become a burden [7,16]. The second strategy, which involves encouraging young adults’ autonomy regarding diabetes management, focuses on identifying the actions that are within the young adults’ own control. The healthcare professionals sometimes purposefully choose not to comment on high measurements or sub-optimal habits in order to emplot a narrative in which young adults can trust their own ability to manage their illness. The third strategy focuses on lowering the standards for diabetes management. By creating standards during the consultation that are easier to achieve, young adults have a better chance of managing their diabetes successfully, which may lead to setting new standards that are higher than the starting point. The third strategy gives young adults a sense of being seen and understood as individuals, which can strengthen the trust between the healthcare professional and the young adult. From a narrative perspective, trust is instrumental in the effectiveness of healthcare, especially in regards to chronic conditions [8]. All three strategies mirror important ways of living with chronic illness during times of transition, which touch upon three aspects: Clinical encounters, daily diabetes management, and emerging adulthood with type 1 diabetes.

### 4.1. Clinical Encounters

In important ways, living with T1D amounts to a form of “surveillance life”, involving lifelong regular interaction with a healthcare system in the form of outpatient check-ups (Heinsen et al., 2022). At such diabetes clinic outpatient visits, the task of self-management and caring for one’s chronic illness is discussed among people living with T1D, their healthcare professionals, and, at times, also relatives in the consulting room. The consulting room serves as a place not only for surveilling how the illness is developing, but also for discussing what actions toward better diabetes practice can be achieved, what patients find important in relation to their illness, and how life with the condition can be improved [7]. In this way, consulting rooms are places where good care is practiced. Good care, according to Mol (2008), is a matter of helping patients find the best solution for their specific situation and responding to the needs they express. The strategy of encouraging enhanced autonomy in diabetes management promotes a feeling among the patients that their life situation is acknowledged.

In the clinical encounter, access to electronic health records in consultations has shifted the focus away from direct interaction with patients in the room and toward the authority of the records and numbers found in electronic health records. The regularized measurement of HbA1c thus acts as an objective indication of progress, which weighs against the subjective experience of living with diabetes [17].

Mattingly (2010) describes how the clinical encounter facilitates a space for healing dramas to take place. Healing dramas stand as a sort of enriched present that portends for different lived life outside of the clinic [8]. Good healers know how to read the situation and respond to the patient’s reactions and needs. When healing dramas occur, it is because the health care professional reads the patient and acts according to their responses, even if it means deviating from treatment plans [8]. The narrative work of a healthcare professional requires a clinical interaction which exceeds looking only at biomedical variables. Itis a form of clinical knowledge learned through numerous interactions and consultation experiences [18]. When clinical encounters are successful, they form trust and portend a better future with illness.

Yet maintaining trust with young adults entails achieving a delicate balance. For the young adults who are leaving the paediatrics department and their long term relationships with the specialist team are often ending [19]. New relationships must be established with the new team of healthcare professionals. Studies on trust between HCPs and patients show that there must be respect for the patient’s autonomy and the professional’s autonomy. Furthermore, there must be a mutual exchange between the healthcare professional and the patient during the clinical encounter in order for a trusting relationship to take root [20]. Trust in healthcare settings is necessary as the patient is in a structurally inferior position and relies on their doctor or nurse in order to get access to certain treatment options [21]. Some of the young adults, like Maja presented in this article, are critical in regard to whom they involves in their diabetes management. Maja has left the consulting room on multiple occasions when she has not felt understood, and she does not hold back when expressing her point of view. In the process of making the young adults feel welcome and safe when they show up for consultations, the healthcare professionals may end up promoting lifelines for diabetes management instead of guidelines, thus differing from the clinical guidelines for treatment.

The consultation room should be a place where good care helps patients find the best solution for their specific situation and responding to the needs they express [7]. The care process around diabetes involves a team of healthcare professionals, machines, patients, bodies, and others, all of whom have a role in diabetes management. Tasks can be shared and can shift with the introduction of a new life circumstance or technology. The consultation thus becomes a space where one can discuss how to improve one’s life with the illness. Illness care does not come in a specific shape or size, but is created in interaction with others, where tasks are negotiated [7]. Starting from patients’ specific situation also means using the consultation to discuss worst-case scenarios regarding diabetes practice. Offering recommendations for how to act when you are drunk and have T1D is a way to start from the young adult’s situation. For healthcare professionals, expertise and tacit knowledge are required to interpret how the specific patient can be helped in the best way [18]. As Malterud (1995) points out, clinical practice requires the healthcare practitioner to understand the particulars in order to negotiate management with the patient and pass on knowledge from groups to the individual. This balance of keeping in mind the universals while integrating the particular patient’s experience leads to constructing illness narratives outside of biomedical variables. In the process of accommodating the needs of a particular patient, healthcare professionals sometimes deviate from clinical guidelines in order to strengthen the relationship and the patient’s illness management.

### 4.2. Daily Diabetes Management

The chronic care infrastructures surrounding diabetes involve a team of healthcare professionals, insulin pumps, monitoring equipment, blood chemistry analysis machines, patients, bodies, and family members (not to mention friends and colleagues), all of whom take part in diabetes management, be it directly or indirectly. Tasks can be shared and can shift with the introduction of new life circumstances or new technologies. The consultation thus becomes a space where one can discuss how to improve life with the illness and where prognostic calibrations take place [11] Illness care does not come in a specific shape or size, but is created in interaction with others, where tasks are negotiated [7] and forms of chronic homework are assigned [14]. Living with T1D also means living with diabetes management technology. Such technologies enhance the possibility to live well with diabetes, but they require knowledge about them and their use before they can become active tools in diabetes management [16]. People with T1D manage their diabetes either with a pen or a pump to deliver insulin as well as a technology to monitor their blood glucose. Most young adults with T1D have been offered a continuous glucose monitor (CGM), which is attached to their bodies and provides ‘real time’ blood glucose levels, while others measure their levels ‘manually’ by pricking their finger and analysing the resulting drop of blood.

Whenever a new diabetes technology is introduced, it changes the way the illness influences everyday life. Technology and knowledge can reconfigure the future [22], and most diabetes technologies produce new knowledge about the illness. The power of a new technology lies in the fact that it replaces existing practices and infrastructures around self-monitoring [23]. At the same time, while technology can improve life with diabetes, patients must have knowledge about and experience with it if it is to be used as an active tool in everyday diabetes management [16]. From a narrative perspective, technologies offer a possibility to reconfigure what a future with diabetes can look like. Everyday use of technology is often discussed in outpatient consultations as a way to ‘tame the technology’ so it does not become too invasive in patients’ everyday life [7].

With daily tools for measuring and monitoring blood glucose, living by numbers has become an important means of managing diabetes. Access to easy measurement tools has changed how one relates to one’s own body and health. Numbers then become a way to shape one’s illness story [24]. This is clear in the case of Peter, who abstains from measuring his blood glucose because he does not want to have to deal with a high number.

### 4.3. Emerging Adulthood with Type 1 Diabetes

Jeffrey Arnett (2000) argues that in industrialized countries, late adolescence (18–25 years of age) is a unique period that should be included neither in adolescence nor in adulthood. During this period, options concerning the individual’s career, love life, and social life are explored, and decisions perceived as determining adult life are made. It is the time in life that contains identity-constituting processes. Having a strong sense of identity has been shown to be an advantage in relation to the outcome of diabetes management among young adults living with T1D [25]. While establishing their future life course, emerging adults orient themselves toward multiple futures. When living with diabetes, they must also come to terms with their own illness and gradually become more independent in their daily diabetes self-management.

For young adults in Denmark, there is a great deal of focus on pressure, perfection, performance, and achievement [26]. Furthermore, Danish youth obtain social capital in particular by participating in a culture of alcohol consumption [27]. Young adults experience pressure in multiple arenas, like their peers without T1D. For emerging adults with T1D, their illness can be an extra stress factor, resulting in a higher frequency of adolescents and young people living with both T1D and anxiety or depression [28]. Many young adults express that their diabetes management, to some extent, is experience-based. This puts healthcare professionals in a situation where, in order to provide good care, they need to acknowledge that young adults are experts on their own life and on making decisions about when to apply good diabetes management and when to prioritize experiences related to youth culture and identity exploration. It is not always easy to make space for diabetes management at a time when young adults are occupied with and worried about their social identities and career decisions. During emerging adulthood, situations arise in which guidelines are disregarded and lifelines for surviving emerging adulthood with T1D are prioritized instead.

The emerging adults have often lived with diabetes for a long time, and the participants in the present study were between 18–21 years of age and had lived with diabetes for 2–18 years. Most of them had had T1D for at least 10 years, and the actions of injecting insulin, counting carbohydrates, and measuring blood glucose were therefore familiar to them. The healthcare professionals must work with a group of young people and create a meaningful framework for diabetes management at a time when the young adults themselves are trying to figure out what their future could look like. Meaning exists in the tension between the future and the past; it is always connected to the uncertainty of the future and can be reconfigured [14]. The task of transitioning out of paediatric care as a young adult also leaves the new healthcare professionals with the task of building trust between them and the young adults while at the same time improving the young adults’ diabetes management.

All three strategies identified in the present article can serve to promote trust while providing lifelines. The technology can be a lifeline for someone like Peter, who was not measuring his blood glucose. Enhanced autonomy can be employed with patients like Thomas to fuel a movement toward integrating better diabetes management to their adult life, or it can be employed to create some kind of path toward any autonomy at all. Realistic standards that depart from the single individual usually set the bar for diabetes management lower than the biomedical guidelines suggest. With the uniqueness connected to emerging adults and their personalities and treatments, the benchmark of a successful consultation varies between simply having patients show up and having them comply with tight regulations, meaning that realistic standards for diabetes care can be placed anywhere in between.

All of this adds up to the view that youth is a special, delimited period in which one is young and experimenting both with illness management and personality. In this process, a division is created between a good diabetes life and a good youth life, and situations such as excessive drinking often force young adults to choose between one or the other. During this period, some things are left unsaid, such as the exact HbA1c target level. Healthcare professionals have also accepted that alcohol consumption is part of young adults’ lives. From a biomedical point of view, combining alcohol consumption and T1D is dangerous, because alcohol limits the body’s own defences against low blood glucose. Nonetheless, healthcare professionals and young adults tend to agree that young adults can participate in alcohol culture if they want to. But if young adults choose to consume alcohol, they are advised on how to limit the danger of combining alcohol and diabetes. For instance, it is a wise strategy to not take insulin at dinner if they know they will be consuming alcohol, even though that advice contradicts regular guidelines for diabetes management. As both young adults and healthcare professionals perceive youth as a specific period, there seems to be an agreement that prioritizing youth life over diabetes life is acceptable so long as some diabetes management actions are incorporated into daily routines. The life stage model suggesting that one fixed stage in life is followed by a liminal period leading to another stable stage [29] argues in favour of treating young adults as a fixed category. Based on this model, the age group of 18- to 25-year-old adults can be expected to grow into managing more steady blood glucose control later in life, as statistics show [3]. Nonetheless, treating an age group as being in a fixed stage between childhood and adulthood is problematic, as it fails to consider the individual processes taking place. Instead, looking at young adults as being at a vital juncture, where the future is largely open for the individual to determine, can be beneficial for understanding the lives of young adults. They are facing institutional changes in the form of graduations, seeking jobs, applying for higher education, and changing clinics, but these decisions are not necessarily made at the same age by all of them.

### 4.4. Strengths and Limitations of the Study

None of the young adults refused to participate, in that they all allowed the researcher to observe their consultation. Still, the population most present at the outpatient clinic is skewed, as youths with dysregulated diabetes are followed more closely than those with well-regulated diabetes and, thus, visit the clinic more often. Moreover, some of the emerging adults with the most severely dysregulated diabetes do not show up for their clinic appointments and were poorly represented in the study. The rather small sample size limits the generalizability of the study findings, yet the data from the young adults and healthcare professionals contain thick descriptions. Ethnographic studies generally do not set out to address general tendencies, hence the focus of the present study has been to investigate social interactions between young adults with T1D and healthcare professionals at a specific outpatient clinic. The three strategies presented in the study were observed in multiple consultations and, when writing up the analysis, these strategies were discussed with some of the practitioners, who confirmed the use of such strategies. This does not mean that all strategies at the outpatient clinic have been explored and presented here or that the strategies cannot be combined or change.

## 5. Conclusions

All three narrative strategies serve to identify and address aspects of diabetes management that cause young adults to feel they are not good enough, and all three try to create experiences that lead young adults to feel they are in control of their diabetes. Thus, the narrative strategies work to emplot a way out of a narrative of failing at one’s own diabetes management. The consultations often end with the healthcare professionals highlighting 1 or 2 points of action that can be addressed moving forward: “try to scan at least every 8 h” or “if we work on taking the insulin at lunch, and preferably prior to eating”. The specific action points give the young adults a feeling that it is possible to move away from negative emotions and narratives about their diabetes management and toward improved management of and a better relationship with their illness. Furthermore, throughout the difficult process of becoming an adult, these strategies serve to promote lifelines for diabetes management by diverting from guidelines at times. In this article, we have iillustrated three strategies through our analysis of consultations with young T1D patients, but they may well be applicable for other young adults with chronic illnesses.

Finally, our studye shows how ethnographic studies in a clinical setting can shed light on the relational work that goes into the clinical encounters. We have presented three narrative strategies that healthcare professionals employ in order to validate the illness work that the emerging adults already perform and at the same time motivate them to optimize their diabetes management. These strategies are especially important when working with a patient population undergoing transitions and identity processes and may be applicable to other chronic conditions and age groups.

## Data Availability

The data presented in this study can be made available on request from the corresponding author, but part are restricted juristically and due to ethical restrictions. All data have been anonymized with respect to the informants and professionals.
